# Systematic Evaluation of Adhesion and Fracture Toughness in Multi-Material Fused Deposition Material Extrusion

**DOI:** 10.3390/ma17163953

**Published:** 2024-08-09

**Authors:** Md Abu Jafor, Neshat Sayah, Douglas E. Smith, Gianni Stano, Trevor J. Fleck

**Affiliations:** 1 Department of Mechanical Engineering, Baylor University, Waco, TX 76798, USAneshat_sayah1@baylor.edu (N.S.);; 2 Department of Mechanical Engineering, Polytechnic University of Bari, 70125 Bari, Italy

**Keywords:** material extrusion, multi-material adhesion, fracture toughness, reptation theory, polymer healing

## Abstract

Material extrusion (MEX) additive manufacturing has successfully fabricated assembly-free structures composed of different materials processed in the same manufacturing cycle. Materials with different mechanical properties can be employed for the fabrication of bio-inspired structures (i.e., stiff materials connected to soft materials), which are appealing for many fields, such as bio-medical and soft robotics. In the present paper, process parameters and 3D printing strategies are presented to improve the interfacial adhesion between carbon fiber-reinforced nylon (CFPA) and thermoplastic polyurethane (TPU), which are extruded in the same manufacturing cycle using a multi-material MEX setup. To achieve our goal, a double cantilever beam (DCB) test was used to evaluate the mode I fracture toughness. The results show that the application of a heating gun (assembled near the nozzle) provides a statistically significant increase in mean fracture toughness energy from 12.3 kJ/m^2^ to 33.4 kJ/m^2^. The underlying mechanism driving this finding was further investigated by quantifying porosity at the multi-material interface using an X-ray computed tomography (CT) system, in addition to quantifying thermal history. The results show that using both bead ironing and the hot air gun during the printing process leads to a reduction of 24% in the average void volume fraction. The findings from the DCB test and X-ray CT analysis agree well with the polymer healing theory, in which an increased thermal history led to an increased fracture toughness at the multi-material interface. Moreover, this study considers the thermal history of each printed layer to correlate the measured debonding energy with results obtained using the reptation theory.

## 1. Introduction

Material extrusion (MEX) has emerged as the most widely used additive manufacturing (AM) method over the past years in a wide range of fields such as automotive, aerospace, and bio-medical [1,2,3]. MEX technology provides several advantages, including cost-effectiveness, reduction in manufacturing time, and simplification of assembly tasks, which have contributed to the rising popularity of the process [4,5,6]. As samples manufactured via MEX AMs are created layer by layer, a weak interlayer strength generally occurs due to many contributing factors, such as poor interlayer contact and insufficient polymer chain diffusion [7,8].

Recent research has attempted to improve the interface material properties and find a solution for increasing interlayer adhesion where process parameters have been studied to optimize experimental protocols and parametric combinations [9,10,11]. Le et al. [12] examined how the printing temperature, infill density, layer thickness, and infill angle affect the tensile strength, elongation, and elastic modulus in TPU-printed samples. Using the Taguchi method to optimize printing parameters, they reported that a 210 °C printing temperature, 45° infill angle, 100% infill density, and 0.1 mm thickness provided the highest tensile strength and the highest elongation at break with 15% infill density. They also reported maximum elastic modulus with 240 °C, 90° infill angle, 100% infill density, and 0.18 mm layer thickness. Arifvianto et al. [13] investigated the effects of printing temperature and raster orientation on tensile strength, elastic modulus, and strain in TPU-printed samples. Five groups of samples were prepared at 0° and 90° orientations, varying printing temperatures between 190 and 230 °C with 10 °C intervals to study the effects of thermal parameters on mechanical properties. They also studied the effects of raster varying the orientations in different angles. According to their test results, a 200 °C extrusion temperature and 0° raster orientation played the most significant role in increasing strength and ductility. Garg et al. [14] studied the influence of layer thickness, infill density, and printing speed to improve the dimensional accuracy of samples printed using TPU. They used analysis of means (ANOM) and response surface methodology (RSM) techniques to show that a 0.2 mm layer thickness, 70% infill density, and 10 mm/s printing speed provided high dimensional accuracy. They also claimed that the layer thickness is the most significant factor for higher dimensional accuracy. Bruère et al. [15] used samples printed with TPU to study the impact of infill orientations and contour lines on tensile properties and stress relaxation behavior. With the test results, they concluded that the contour lines and alternate orientations (0°–90° and 45°–135°) played significant roles in the part integrity and strength of the samples. Goh et al. [16] studied the process–structure–property (PSP) relationships for mode I interlaminar fracture toughness with DCB samples printed with different printing parameters (nozzle temperature, print speed, and bed temperature) using continuous carbon fiber-reinforced nylon. The effect of printing parameters on porosity and fiber directionality was investigated to show that mode I fracture toughness is highly affected by porosity and inter-layer bonding time. It was reported that a higher printing speed caused higher porosity, leading to a low mode I fracture toughness. On the other hand, a higher printing speed increased the toughness due to longer bond formation time. The findings suggested a low print speed, high nozzle temperature, and high bed temperature for high mode I interlaminar fracture toughness. Kariuki et al. [17] investigated three-point bending tests in short fiber-reinforced nylon for flexural behavior in printed samples. The parameters of interest were printing temperature, print speed, layer thickness, and build orientation. Based on the results, all the parameters had a noteworthy influence on the flexural strength and flexural modulus, with build orientation being the most influential parameter. Zhang et al. [18] also used short carbon fiber-reinforced nylon to study the effect of raster pattern and build orientation on the mode I fracture toughness and effective fracture energy of printed samples. A significant effect of build orientation on the fracture toughness of the tested samples was found, but the effect of the raster pattern was reported to be negligible. However, both the studied parameters showed a considerable influence on the effective fracture energy. León-Becerra et al. [19] investigated the impact of printing orientation on the viscoelastic properties and surface roughness of samples using short carbon-reinforced nylon, Onyx, varying the printing orientation in flat, on-edge, and upright positions. In the experiments, they found variations in the glass transition temperature, storage modulus, and loss modulus depending on the printing orientations. They also reported higher roughness values in samples printed in the upright direction.

One of the attractive features of MEX technology is the possibility of extruding two or more materials during the same print and even within the same component, taking advantage of multi-material machines. In this way, smart objects and functionalized parts can be monolithically fabricated without requiring a human assembly task [20,21]. The key enabler to using the multi-material MEX approach for mass production is the quality of the interface adhesion between the two extruded materials. Yin et al. [22] used a multi-material system with TPU and acrylonitrile butadiene styrene (ABS) to study the contribution of the nozzle and build plate temperatures and print speed on the bonding mechanism and mechanical properties of the multi-material structure. They also developed a heat transfer-based model to obtain the temperature profile at the interface to numerically predict the bonding strength. Both their numerical and experimental results indicated an increased build plate temperature to be the most influential parameter on the high bonding strength at the interface. Yavas et al. [23] studied multi-material lattices with polylactic acid (PLA)–thermoplastic polyurethane (TPU) to investigate stiffness, strength, and energy absorption capacity with fracture and flexural behavior tests. They reported the dependency of the microstructural features on the building direction that helped improve interfacial bonding. They also reported the influence of enhanced interfacial adhesion between PLA and TPU on the flexural modulus and energy absorption capacity of the multi-material structure. Altuntas et al. [24] focused on multi-material composites via suture morphology to create more rigid and robust interfaces with varying overlap distances in samples printed with PLA and TPU. They used the DCB test to evaluate the mode I fracture toughness of the interface without any geometric manipulation. In comparison to the baseline interface, their suggested sutural interfaces could increase the interfacial toughness up to 16–18 times. Rabbi and Chalivendra [25] used PLA and nylon in a multi-material structure for mode I and mixed-mode fracture investigation with asymmetric DCB and single-leg bending samples heat-treated by annealing at different temperatures and treatment durations. They reported that annealing under melting temperature reduced the mode I fracture toughness significantly compared to the pristine samples. On the other hand, annealing above the melting temperature with prolonged treatment duration increased the intermolecular diffusion and entanglement process, leading to enhanced fracture toughness. These conditions were consistent with the mixed-mode fracture toughness. Goh et al. [26] investigated the significance of mechanical interlocking and surface roughness at the interface on the increase in adhesion in multi-material structures using conductive PLA and TPU. They correlated printing sequence and interface modifications with surface roughness that resulted in better adhesion. Brancewicz-Steinmetz et al. [27] studied the bond strength between PLA and TPU by varying printing parameters, surface patterns, and material print order. They printed two coaxial cylinders for a shear strength test and investigated the samples for surface roughness and adhesion between polymer layers. The results indicated that surface patterns and printing order had a substantial impact on adhesion strength because of the surface roughness of the material contact surface. Brancewicz-Steinmetz et al. [28], in their other work, again used PLA and TPU multi-material structures with different processing techniques such as post-processing with acetone immersion and annealing, and in situ surface activation with acetone and tetrahydrofuran to improve the bonding between the multi-material interfaces. They recommended the use of tetrahydrofuran solvent because of its lower influence on sample degradation, which is common in other treatment techniques. Since the MEX process is capable of extruding materials with different moduli, it can be used to fabricate bio-inspired structures made up of both soft and stiff materials, taking inspiration from nature. AM technologies have revolutionized the soft robotics field where the manufacture of soft–stiff structures is a key requirement; at the state-of-the-art, MEX seems to be the best manufacturing technology for the fabrication of bio-inspired soft robots [29,30,31].

Although numerous studies on single materials and a handful of works on multi-material structures have already been performed to increase the adhesion between the layers and interface and to improve the mechanical behavior of material extruded parts, in situ processing techniques are still not adequately explored. Additionally, many of the underlying mechanisms that are driving the increased adhesion performance have been left undetermined. In addition to furthering the knowledge of adhesion, multi-material structures require further investigation as the behavior of the structures depends on diverse mechanical properties and rheological characteristics of the materials involved, variability in the printing parameters, complex bonding mechanisms at the interface between two or more materials, and many unexplored factors. To overcome this knowledge gap and further investigate adhesion between soft and stiff materials prepared via multi-material MEX technologies for soft robotics applications and assembly-free structures [5,6], this paper systematically explores the adhesion between a soft material (TPU) and a stiff material (CFPA), showing how selected print parameters and printing artifacts improve interface bonding. The mode I fracture method has been adopted to determine the toughness at the interface of the two materials using the DCB test. To determine the effect of the thermal history on the resultant adhesion, infrared (IR) thermography was used to correlate the reptation time with the bonding energy. The internal microstructure was analyzed with X-ray CT to visualize the porosity level at the interface between the two materials. The findings of the present paper lay the foundation for a more comprehensive exploitation of MEX technologies for the fabrication of bio-inspired structures composed of soft and stiff segments.

## 2. Materials and Methods

### 2.1. Materials

This study aims to evaluate the adhesion between stiff and soft materials extruded in the same manufacturing cycle via MEX technology and assess the influence of various process parameters to maximize the interface bonding. Two commercially available materials were used for this purpose: (1) a thermoplastic polyurethane (TPU 95A; Ultimaker B.V.; Utrecht, The Netherlands) served as the soft material, and (2) a carbon fiber-reinforced nylon (CFPA; MatterHackers Inc.; Lake Forest, CA, USA) served as the stiff material. 

### 2.2. Adhesion Evaluation

#### 2.2.1. Sample Preparation

**Figure 1:**
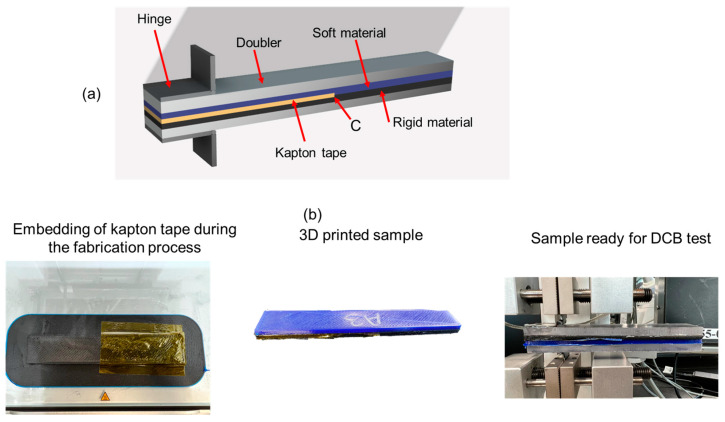
(**a**) CAD design of the sample to be tested to evaluate the multi-material adhesion between soft and stiff materials, (**b**) workflow of the present research from fabrication to testing.

An Ultimaker S3 (Ultimaker B.V.; Utrecht, The Netherlands) equipped with two extruders capable of printing two materials in the same cycle was used to manufacture double cantilever beam (DCB) samples. Figure 1 shows a representative sample geometry and the workflow. All the samples had two parts, each part having the same dimensions with 140 mm length, 25.4 mm width, and 3.25 mm thickness (6.5 mm thickness for both parts in a single sample). Because of the low stiffness of TPU 95A, the samples were stiffened with doublers (or supports) on both sides, according to [32]. Polycarbonate sheets (Plaskolite LLC.; Columbus, OH, USA) with a thickness of 6 mm with the sample geometry shown in Figure 1a were used as doublers to prevent excessive deflection of the beam samples during the test. Thickness of the doublers was selected based on a trial-and-error approach with 2 mm, 4 mm, and 6 mm sheets. The 6 mm thick sheets were found to provide the required capability for preventing the excessive beam deflection. Each sample was composed of parts, each made exclusively of CFPA and TPU, which were printed with the settings appearing in Table 1. Point C in Figure 1a indicates the point of interest for monitoring the thermal history of the printed samples, as described later in Section 2.3 and Section 3.2. A 0.06 mm thick Kapton film (Advanced Polymer Tape Inc.; Newmarket, ON, Canada) of 76 mm length from one edge of the sample was placed between layers 13 (the final layer of the first material) and 14 (the first layer of the second material) to create a pre-crack for the testing process. Figure 1b represents the workflow for the work from fabrication to testing. Using a custom gcode command, the printing process was paused after depositing layer 13 for a specified amount of time, during which the Kapton tape was manually embedded before resuming the printing process, as shown in Figure 1b. After manufacturing, the doublers were glued on both sides of the samples, and afterward, the piano hinges (50.8 mm overall width) were attached over the doublers using an epoxy adhesive (Loctite EA 9309NA Aero; Henkel Corporation; Rocky Hill, CT, USA) and held for six hours at 80 °C for curing.

#### 2.2.2. Design of Experiment (DoE) for Multi-Material Adhesion

**Figure 2:**
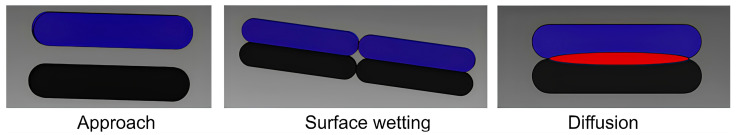
Schematic diagram showing the polymer healing steps between the soft and stiff materials.

Process parameters are vital in defining the final mechanical properties of structures fabricated through an MEX process. The general healing model for fracture stress at the material interfaces proposed by Wool and O’Connor [33,34] is expressed in Equation (1) [35].

(1) $$\frac{\sigma }{\sigma_{\propto }}=\left[\frac{\sigma_{0}}{\sigma_{\propto }}+\frac{K}{\sigma_{\propto }}\cdot \;t^{\frac{1}{4}}\cdot \psi \left(t\right)\right]\cdot \;\varphi (t)$$

where $\sigma_{\propto }$ is the strength of the bulk materials [MPa]; $\sigma_{0}$ is the interfacial strength due to wetting [MPa]; *K* is a constant proportional to one-fourth power of the polymer diffusion coefficient; *t* represents time [s]; $\psi \left(t\right)$ is a dimensionless diffusion initiation function; and φ(t) is a dimensionless wetting distribution function. The model is governed by the polymer healing steps which are shown in Figure 2.

In this work, the adhesion between the soft and stiff materials was systematically studied by investigating the effect of three selected process parameters (and their interaction) focusing on the two significant functions in Equation (1), the wetting function, φ(t) and the diffusion function, $K\;and\;\psi \left(t\right).$ These are controlled by the surface wetting and diffusion of polymer healing, as in Figure 2, which is dependent on many factors such as thermal history, pressure, and polymer chain length. In the polymer healing process, the molten material is extruded on the previously deposited material, and the thermal energy of the freshly deposited material causes surface wetting between the boundary of the two materials. Because of adequate surface wetting, the polymer chains of the two materials diffuse across the boundaries to create bonding at the interface by polymer chain entanglement. By providing more thermal energy or reducing thermal gradients between the beads, it is possible to facilitate surface wetting that can result in more diffusion creating stronger bonds at the interface with more entanglements. Additionally of note, non-rectangular bead shapes during the material deposition create inter-bead voids or porosity that compromise the mechanical properties of the end parts. Based on this understanding, to improve the adhesion between the multi-materials, three process parameters were selected for this study that included material print order (MPO), use of a hot air gun (HA), and ironing (I), with two levels (low and high) for each parameter. The low level (−1) was considered when the CFPA part was printed at the bottom, and ironing and the hot air gun were OFF. On the other hand, the high level (+1) was considered when the TPU part was printed at the bottom, and ironing and the hot air gun were ON. A factorial plan 2^3^ was used with a number of repetitions equal to five for every combination. These parameters of interest for the design of an experimental study with selected levels to manufacture a total of forty test samples with the different combinations investigated can be seen in Table 2.

**Figure 3:**
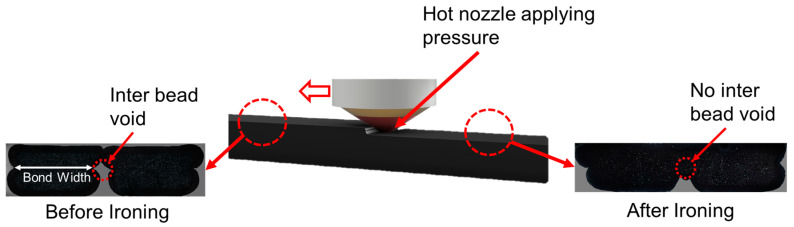
Representative schematic of the ironing process: it should be noted that the hot nozzle passes over the last extruded layer without extruding materials—mechanical pressure is applied.

Ironing was selected as a means of reducing the porosity/voids, potentially increasing the wetted surface area at the interface (cf. Figure 2). The external hot air gun was used to increase the thermal energy and provide a longer time for the polymer chain diffusion with a focus on improving the adhesion between the multi-material. Test samples were manufactured in a random order as determined by the software Minitab 17 (Minitab, LLC.; State College, PA, USA) to mitigate the effects of the uncontrollable external factors related to the manufacturing process, such as vibrations, changes in room temperature, and humidity. A brief overview of the three process parameters is provided, as follows:

- Material Print Order (MPO): The material print order signifies the position of the materials in the printed samples. In four of the eight combinations, CFPA was placed at the bottom, and in the other four, the bottom layer was TPU 95A. As the pressure of the material being applied is known to affect interlayer adhesion, the material printing order and, therefore, the resistance of the deposition surface may influence the adhesion process.
- Ironing (I): Ironing is a process where mechanical pressure is applied with the extruder on the deposited material layer, which in turn exerts pressure on the interface between the top two beads to increase the adhesion between, in this case, the soft and stiff materials. Figure 3 provides a schematic of the ironing process. During the ironing process, the extrusion material is retracted back into the nozzle, stopping the extrusion process such that the nozzle tip is only used to apply pressure to the top surface of the previously deposited beads. Ironing seeks to achieve benefits similar to mechanical rollers, which have been shown to increase interlayer adhesion without the need for extra hardware or printer modification. As can be seen in Figure 3, inter-bead voids are created due to the non-rectangular shape of the beads which can be reduced by applying mechanical pressure with the ironing process. This helps in increasing the bond width and subsequently increases the inter layer adhesion. In this study, ironing was performed over the area where there was no Kapton tape and was only applied on the first layer of the second material (14th layer). Ironing is a built-in feature available in the slicing software (Ultimaker Cura 5.4.0). Table 3 contains the parameters used in the ironing process.

- Hot air gun (HA): A hot air gun was used to manipulate the manufacturing environment by raising the temperature of the print surface. When the printing with the lower material was complete, the print process was stopped automatically using the “pause” command in the gcode to allow for the placement of the Kapton film prior to depositing the first layer of material 2. The hot air gun was turned on when the build plate started moving from the halted position, and its motion continued for two minutes. Hot air was applied near the end of the film at the section that was not covered by the Kapton film. When the extruder started depositing material 2 on the uncovered section, the hot air supply was stopped.

#### 2.2.3. Mechanical Testing Procedure

**Figure 4:**
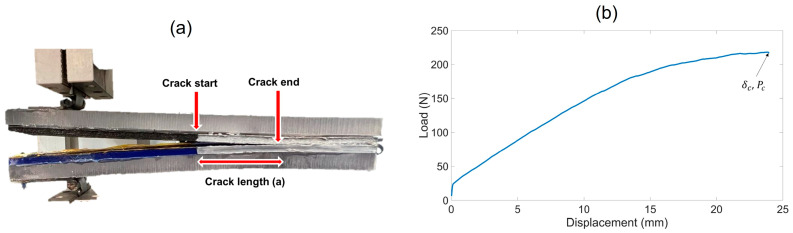
(**a**) Setup for DCB test, (**b**) representative load–displacement curve.

DCB samples were tested using a TestResources DG.1000 dynamic testing machine (TestResources, Inc.; Shakopee, MN, USA) instrumented with a 4448.2 N load cell. The long edges of the samples were coated with a layer of water-based white typewriter correction fluid to obtain a precise visual detection of the crack propagation. The samples were mounted in the dynamic testing machine horizontally, and the doublers ensured that the samples remained horizontal throughout load application, as shown in Figure 4a. The use of doublers can contribute to the fracture toughness because of the increased overall sample thickness, possible damage developed, and delamination between the actual sample and doubler interfaces. This effect of doublers can be eliminated following a similar approach as [36]. However, in this work, no data reduction method was used to eliminate the effect of doublers; rather, a simplified approach was adopted to run the tests and calculate fracture toughness to investigate the influence of print parameters and printing artifacts. A consistent testing procedure was followed for all the samples and any sample with damage/delamination in the sample–doublers interfaces was not considered in the calculation. All samples were tested in ambient room temperature and loaded at a 2 mm/min crosshead displacement rate with a data acquisition rate of 10 Hz for 12 min. Therefore, all the tests were run until the crosshead displacement approximated 24 mm, and the load time was recorded as a function of time. Load-displacement values of the tensile test machine were recorded every 0.1 s from XY 4.0 series software (TestResources, Inc.; Shakopee, MN, USA) using a Q controller. Figure 4b shows a representative load–displacement curve. The crack growth was recorded using a Dino-lite Edge 3.0 (Dunwell Tech Inc.; Torrance, CA, USA) digital microscope placed perpendicular to the crack growth direction. With the data obtained from the DCB test, mode I fracture toughness, $G_{IC}$, was calculated using the unmodified beam theory [37]:

(2) $$G_{IC}=\frac{3\;P_{c}\delta_{c}}{2ba}$$

where, $P_{c}$ is the critical force for mode I fracture [N]; $\delta_{c}$ is the crosshead displacement at the critical load [mm]; *b* is the width of the DCB sample [mm]; and *a* is the crack length [mm].

### 2.3. Thermal History Monitoring

A thermal camera (FLIR A700, Teledyne FLIR LLC.; Wilsonville, OR, USA) equipped with a Macro lens of 2.0× magnifying factor captured videos for infrared (IR) thermography to evaluate the temperature profile of each sample during the manufacturing process. The camera has a resolution of 640 pixels by 480 pixels with 24 μm/pixel with a manufacturer-rated precision of ±2 °C. Videos of part surface temperature were recorded at a frame rate of 30 Hz. Non-uniformity corrections (NUC) were made using the automatic NUC function in the camera software, FLIR Research Studio 2.1. The camera was placed normal to the x–y plane (cf. Figure 1b) of the printer, focusing on the end of the Kapton film at point C in Figure 1a. The cursor point of the FLIR A700 was placed at the end of the Kapton film for a 1-pixel measurement to record the temperate history of that point in the test sample as the print progressed. The temperature profile for point C in Figure 1a was recorded from the instance in which the material was deposited at this location through the end of the print. Data were exported from the temperature profile to capture the thermal history of the point of interest in each layer of the sample section printed with the second material.

### 2.4. Microstructure Analysis via X-ray CT

Among all test samples appearing in Table 2, combinations A and H were found to have the highest and lowest fracture toughness, respectively, as shown in the later sections. Therefore, the microstructure of each of these two combination samples was further evaluated using an NSI X3000 X-ray µCT system (North Star Imaging Inc.; Rogers, MN, USA) with a resolution of 10 microns. This level of resolution is infeasible when the objective is to scan the entire part of a relatively large sample such as that studied here. Therefore, each sample was divided into two regions of interest (ROI) along the print direction, and samples remained unchanged along other directions to evaluate the whole part’s microstructure. µCT images were generated by setting the X-ray source at 65 kV and 900 µA. Each ROI was rotated 360 degrees during the scan, and 2100 projections were developed. The efX-CT 2.4 reconstruction software (North Star Imaging, Inc.; Rogers, MN, USA) was then employed to reconstruct the scan data. The reconstructed data were then imported into the VGStudio Max 3.4 software (Volume Graphics GmbH; Heidelberg, Germany) to evaluate the void content within the microstructure of each scanned ROI. The VGStudio Max porosity analysis module was utilized to assess the voxel data set for voids within the microstructures of the ROI [38,39].

## 3. Results and Discussion

### 3.1. Design of Experiments for Inter-Material Fracture Toughness

**Figure 5:**
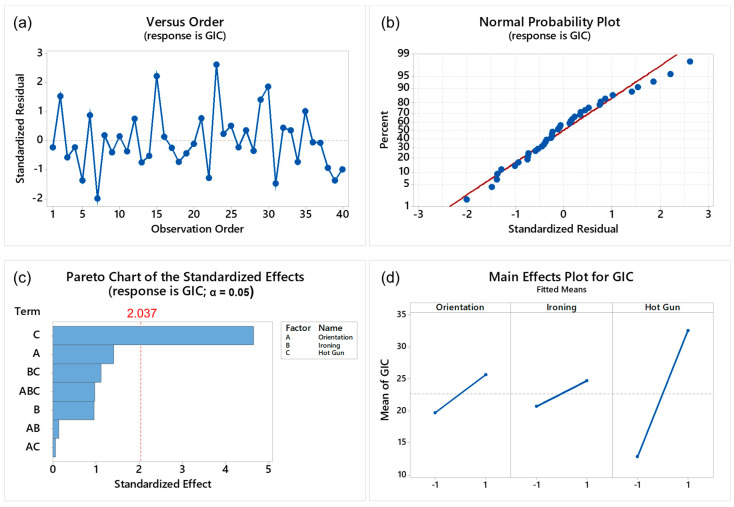
DoE *G_IC_* material print order residuals analysis with (**a**) residual values as a function of test order, (**b**) residual normal probability and the results obtained from the DoE, (**c**) Pareto chart of the standardized effects in which the response is *G_IC_* (*α* = 0.05), and (**d**) main effect for each parameter considered.

The purpose of the DoE described above was to determine the set of process parameters that have the most influence on adhesion at the interface between stiff (CFPA) and soft (TPU) materials extruded in the same manufacturing cycle. The fracture toughness is used here as a metric to evaluate the adhesion, where an increase in fracture toughness is used to indicate an increase in the interfacial adhesion. A residual analysis was also performed to show that the DoE was not negatively affected by the material printing order or unwanted environmental conditions. Figure 5b shows that the residual data are normally distributed and uncorrelated with the observation order, as shown in Figure 5a. Table 4 contains the average $G_{IC}$ values and their standard deviations for the eight combinations tested. These results show that with the inclusion of each studied parameter (MPO, I, HA), the average fracture toughness has increased significantly, with combinations A and H having the lowest and highest parameter values, respectively. The test samples from combinations A, D, and H showed the most consistent results with smaller standard deviations; however, combination G had the highest standard deviation as one sample showed an unexpectedly high fracture toughness value. Figure 5 also shows the contribution of every factor analyzed in the DoE on interface adhesion. The standardized effect plot in Figure 5c shows the effect of each factor and the factor interactions on $G_{IC}$, while the mean effect plot in Figure 5d shows the effect produced by the low and high levels of every factor.

In particular, the following conclusions can be drawn from the DoE analysis:

- The hot air gun parameter is the only process parameter providing a statistically significant effect on increasing the interface adhesion (increasing of $G_{IC}$). It produces a standardized effect of 4.73, which is significant given the threshold effect of 2.037 (cf. Figure 5c). When switching from the low level (hot air gun OFF) to the high level (hot air gun ON), the mean $G_{IC}$ moves from 12.3 kJ/m^2^ to 33.4 kJ/m^2^, as shown in Figure 5d.
- The MPO and the ironing process parameters do not produce a statistically significant effect on adhesion, as shown in Figure 5c, in which the standardized effects of both cases are lower than the threshold effect (2.037). However, ironing played an influential role in reducing porosity which can be seen in the later section. Also, the interaction among all three studied process parameters has no remarkable effect.

From the analysis above, the only process parameter among those considered here that has a significant effect on interface adhesion is the usage of the hot air gun, which is in agreement with [7,35]. An increase in the temperature between layer *k* (made up of TPU, or vice versa) and layer *k* + 1 (made up of CFPA, or vice versa) increases the intermolecular polymer chain diffusion, which generates stronger interlayer bonds between the two materials and thus promotes adhesion. It is worth mentioning that the material printing order (soft material at the bottom, stiff material at the top, and vice versa) and the ironing process parameters do appear to have a significant effect on the intralayer polymer diffusion chain. Due to the different chemical compositions of the two materials (stiff and soft), the applied mechanical pressure alone did not produce a significant increase in interfacial bonds in this study. It should be noted that, despite high values of standard deviations, mainly due to the high variability affecting MEX process, the DoE is consistent, as the *p*-value of the hot air gun parameter is lower than 0.001.

**Figure 6:**
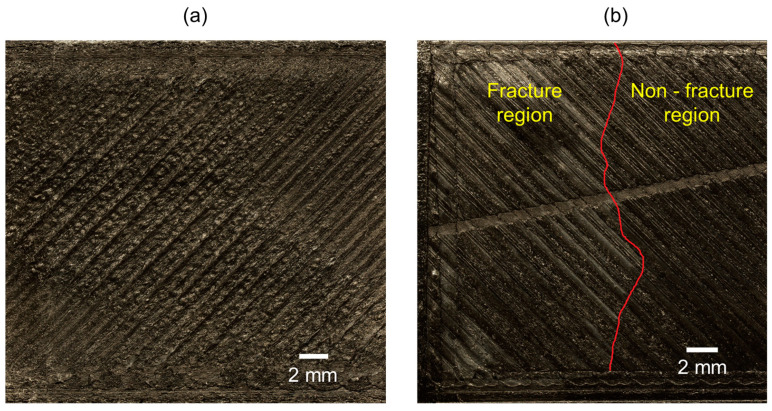
Fracture surfaces with (**a**) combination A (CFPA bottom), (**b**) combination H (CFPA top) with red line indicating separation of fracture and non-fracture regions.

Figure 6 represents the fracture surfaces in CFPA parts for combinations A and H based on the lowest and highest values of $G_{IC}$. A Keyance VHX-7100 microscope (Keyence Corporation; Osaka, Japan) with 20× magnification was used to capture the images. In both cases, the residue of the other material (TPU) was not seen, indicating the fracture occurred at the interface, not in the material interlayer. Figure 6a shows the fracture surface of combination A, in which any traceable boundary to separate the fracture region could not be identified, indicating a larger fracture area. However, as shown in Figure 6b, combination H had a clear indication of the fracture region which was separated by the red boundary. The smaller fracture region indicates a smaller crack length that resulted in higher fracture toughness at an increased load. Also, the non-uniform fracture region indicates non-uniform crack propagation due to the enhanced bonding between the two materials. Additionally of note, the reduced interbead ridges seen in Figure 6b can be attributed to the lower agglomeration of materials in the grooves due to the use of ironing, which in turn reduced the porosity at the interface, as discussed in the later sections.

### 3.2. Thermal History Results

**Figure 7:**
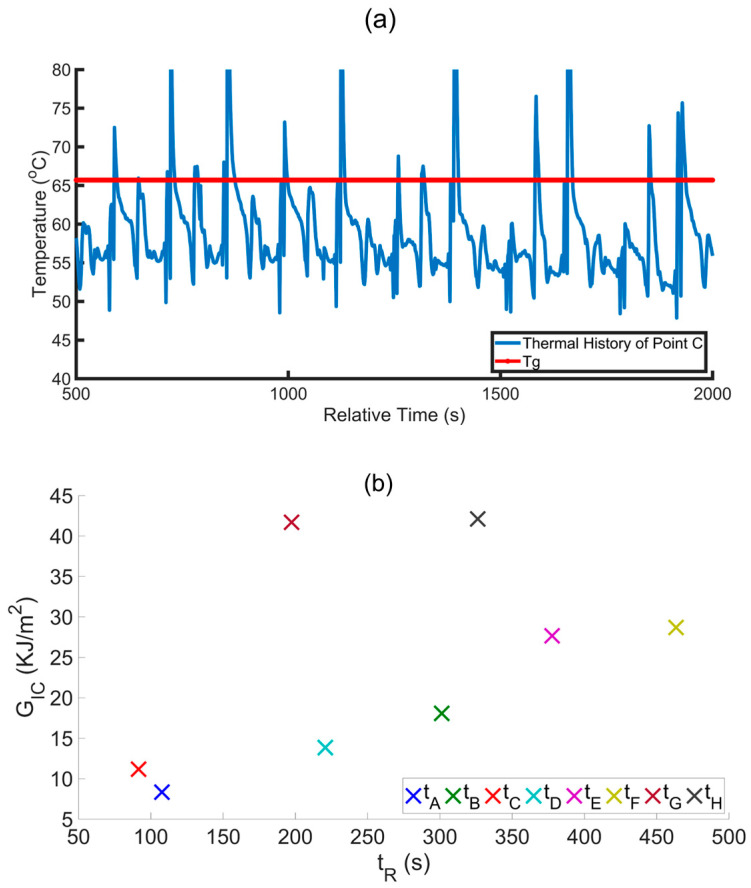
Temperature history effect on fracture toughness. (**a**) Typical temperature vs. relative time for the total printing time at point C [cf. Figure 1a], (**b**) fracture toughness *G_IC_* as a function of *t_R_* for all test samples.

Time-dependent temperature variation profiles in each layer of the printed samples were extracted to investigate further the correlation between reptation time and $G_{IC}$ value. The thermal history of the interface between the two investigated materials was monitored following deposition to assess the polymer healing at the interface in our multi-material MEX structures [33]. For polymer healing in multi-material structures, the molten material of one type is extruded on the previously deposited material of a different type. Due to the thermal energy of the second deposited material, surface wetting takes place between the boundary of the two materials. The polymer chains of the two materials then diffuse with each other to create bonding at the interface by polymer entanglement (cf. Figure 2). Figure 7a shows a typical temperature–time plot where it can be seen that the print temperature exceeds the glass transition (*T_G_*) temperature of CFPA (65.7 °C) for a significant amount of time during deposition. The point of interest for each of the eight samples is at point C (cf. Figure 1a), which was tracked throughout the print. To this end, point C for each deposited layer was tracked by shifting the pixel of interest to record the temperature profile of each layer. All the temperature profiles were then shifted in time to align the starting point of initial material deposition at time *t* = 0, which is used to define the relative time in Figure 7a. The reptation time *t_R_* is the total amount of time that the temperature of a material point remains above *T_G_* [35,40,41]. As the printing temperature and printing speed for CFPA were higher than the TPU, the total time and, therefore, the thermal history were not constant for all samples. Figure 7b illustrates the relationship between the *G_IC_* and *t_R_*, showing that combination E and combination F, which used the hot gun, have higher *t_R_* values as the temperature at the interface remains higher for a longer time. In addition, combination G and combination H show higher bonding strength because of the effect of both ironing and the hot gun. However, due to the extended time required for the ironing process, the *t_R_* values are comparatively lower than those of combination E and combination F.

### 3.3. Microstructure Results

**Figure 8:**
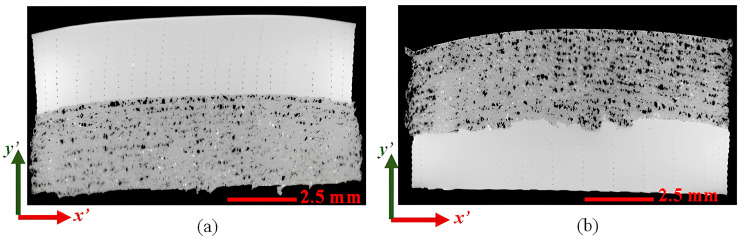
X-ray µCT 2D top cross-section with (**a**) combination A (TPU top, CFPA bottom), (**b**) combination H (CFPA top, TPU bottom).

As stated in Section 2.4, combinations A and H were further evaluated based on the minimum and maximum average $G_{IC}$ values using the NSI X3000 X-ray µCT system (North Star Imaging Inc.; Rogers, MN, USA) system to better understand the microstructure of the samples near the material interface. The 2D cross-sections of combinations A and H appear in Figure 8a,b. As shown in Figure 8a, the interface between CFPA and TPU is smooth and recognizable in combination A. Alternatively, the material interface is unsmooth and quite irregular in combination H, as shown in Figure 8b, most likely due to the use of ironing during the printing process. The noted effect was demonstrated in the literature [42]. It should also be noted that this increase in adhesion for combination H could not only be a thermal effect that enabled the interface to remain above the glass transition temperature for a longer period due to the use of the hot air gun but additionally due to mechanical interlocking at the interface that can be seen at the interface in Figure 8b.

**Figure 9:**
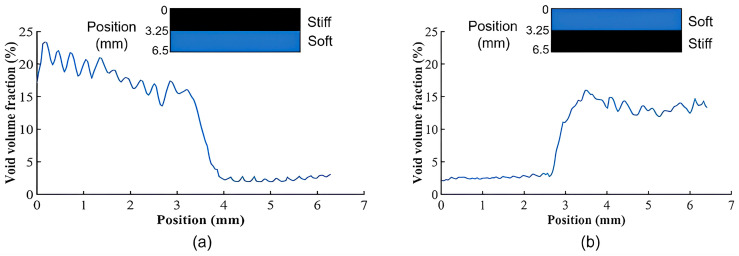
Void volume fraction along the y’-coordinate direction of (**a**) combination A, (**b**) combination H.

The void volume fractions along the y’-coordinate direction (cf. Figure 8) within combinations A and H are shown in Figure 9a,b. Note that, in both combinations A and H, the z’-direction is the extrusion direction, while the y’-direction is perpendicular to the print plane. The void volume fractions were calculated using an average of 10 points along the interface of each sample. As shown in Table 5, the void volume fraction is 6.86% at the interface in combination A, while the void volume fraction is 5.08% at the combination H interface. Moreover, the overall average void volume fraction computed from the data in Figure 9 is 11.30% within the microstructure of combination A and 8.57% within the microstructure of combination H, which indicates that the use of ironing and a hot air gun during the printing process caused a reduction of 24% in the overall average void volume fraction. This reduction is likely to contribute to the increase in fracture toughness between these two combinations, which would lead to fewer stress concentrations acting as crack initiation points.

### 3.4. Summary of Results

**Figure 10:**
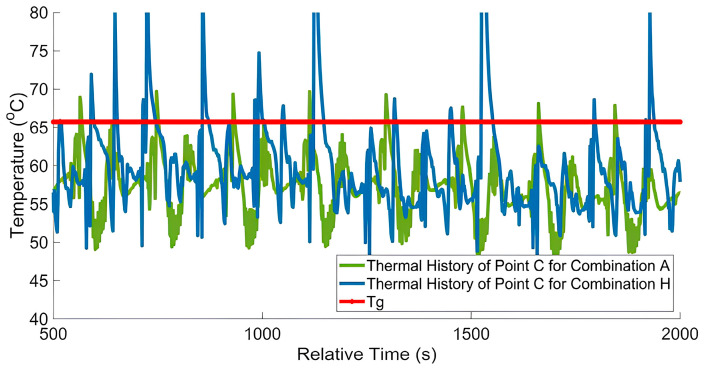
Comparison of temperature vs. relative time between combination A and combination H for the total printing time at point C [cf. Figure 1a].

The goal of this work was to demonstrate simplified approaches to increase adhesion in multi-material structures and further understand which aspects of the deposition process led to improved adhesion between dissimilar materials. As mentioned in the previous sections, the hot air gun was used to provide increased thermal energy allowing the multi-material interface to remain above the glass transition temperature for an extended period that facilitated polymer chain diffusion, which resulted in the most significant effect for improving fracture toughness. Figure 10 shows the comparison of the temperature histories of combination A with combination H, and the scenarios with the highest and lowest resultant fracture toughness, respectively. As can be seen for combination H, the temperature at the multi-material interface (i.e., see point C in Figure 1b) not only remained above *T_g_* longer, but achieved higher temperatures overall as compared to combination A. It is believed this enhanced thermal history leads to improved polymer chain diffusion, resulting in higher fracture toughness for combination H.

It should be noted that the ironing process is expected to have had less of an effect on improving the fracture toughness from combination A to combination H. Instead, it is expected that the mechanism for enhancing fracture toughness in combination H is more likely due to having a reduced porosity and a higher potential mechanical interlocking, as shown in Figure 8b. The highest fracture toughness exhibited in this work occurred when both the hot gun enhanced the thermal history, and the ironing reduced the overall porosity. A comparison of these highest and lowest fracture toughness print scenarios is highlighted in Table 6.

## 4. Conclusions

This work investigated the interfacial adhesion in multi-material structures manufactured by MEX. The mode I interlaminar fracture toughness was employed to measure the debonding energy in the multi-material interface. Three process parameters, material print order, ironing, and hot air gun use, were investigated in eight combinations to gain insights into their influences on the structural performance of the MEX-printed multi-material structures. The statistical results based on a total of forty samples showed that the hot air gun had a significant effect on the increase in fracture toughness between the two materials, whereas the effects of material print order and the ironing process were less significant. The test results also showed that interaction among all three studied process parameters did not produce a remarkable effect on the interface adhesion. IR thermography was also conducted on the printed samples to monitor the thermal history to correlate between the time above glass transition temperature at the interface and the debonding energy of the material interface in light of polymer healing theory. Combinations E and F, which employed hot guns, had the highest *t_R_* values among the eight combinations because the temperature at the contact was higher for a longer period. However, due to the combined effects of the heat gun and ironing, combinations G and H exhibited stronger bonds. X-ray µCT was also used in this work to measure the variation in porosity through the sample and at the interface in the test samples. A significant reduction in average void volume fraction was identified in the samples that used both ironing and the hot air gun, which correlated well with increased fracture toughness. In addition to observing a reduction in porosity, the X-ray images showed irregularities due to ironing at the material interface, which could lend itself to mechanical interlocking as an additional adhesion mechanism. 

## Figures and Tables

**Table 1:** Pertinent printing parameters.

Material	CFPA	TPU 95A
Nozzle Temperature	260 °C	227 °C
Print Speed	35 mm/s	25 mm/s
Nozzle Diameter	0.8 mm	
Bead Width	0.8 mm	
Layer Height	0.25 mm	
Build Plate Temperature	70 °C	

**Table 2:** Process parameter combination naming convention.

Combination	Material Print Order	Ironing	Hot Air Gun
A	−1	−1	−1
B	+1	−1	−1
C	−1	+1	−1
D	+1	+1	−1
E	−1	−1	+1
F	+1	−1	+1
G	−1	+1	+1
H	+1	+1	+1

**Table 3:** Ironing parameters.

Parameters	Values
Pattern	Zigzag
Line Spacing	0.4 mm
Flow	0%
Inset/Distance from the edge	0.8 mm
Speed	10 mm/s

**Table 4:** Average fracture toughness values for the eight combinations.

Combination	$\mathrm{Average}\;G_{IC}$	Standard Deviation
A	8.34	4.89
B	18.08	11.21
C	11.18	8.11
D	13.85	5.23
E	27.67	13.42
F	28.70	15.82
G	41.70	43.31
H	42.12	6.83

**Table 5:** Void volume fraction with combinations A and H.

Sample	Void Volume Fraction in CFPA	Void Volume Fraction in TPU	Void Volume Fraction at the Interface	Average Void Volume Fraction
Combination A	17.6%	2.5%	6.8%	11.3%
Combination H	13.1%	2.6%	5.1%	8.6%

**Table 6:** Summary of results.

	Worst Parameter—Combination A	Best Parameter—Combination H	Improvement
Fracture Toughness [kJ/m^2^]	8.34	42.12	405%
Relative Time [s] (Time above *T_g_*)	108	326	202%
Void Volume Fraction (%)	11.3	8.6	24%

## Data Availability

The original contributions presented in the study are included in the article, further inquiries can be directed to the corresponding author.
